# The Effect of Effort During a Resistance Exercise Session on Glycemic Control in Individuals Living With Prediabetes or Type 2 Diabetes: Protocol for a Crossover Randomized Controlled Trial

**DOI:** 10.2196/63598

**Published:** 2024-11-05

**Authors:** Marissa Ramirez, Maja Gebauer, Christine Mermier, Jonathan Peter Little, Luotao Lin, Gabriel Palley, Yu Yu Hsiao, Roberto Ivan Mota Alvidrez, Zach A Mang, Fabiano Trigueiro Amorim, Valmor Tricoli, Flavio De Castro Magalhaes

**Affiliations:** 1 Department of Health, Exercise & Sports Sciences College of Education and Human Sciences University of New Mexico Albuquerque, NM United States; 2 School of Health and Exercise Sciences University of British Columbia – Okanagan Campus Okanagan, BC Canada; 3 Department of Individual, Family, and Community Education College of Education and Human Sciences University of New Mexico Albuquerque, NM United States; 4 Department of Family and Community Medicine Health Science Center University of New Mexico Albuquerque, NM United States; 5 Clinical and Translational Sciences Center Pharmaceutical Sciences Department University of New Mexico Albuquerque, NM United States; 6 Albuquerque Baseball Academy Albuquerque, NM United States; 7 Department of Sport School of Physical Education and Sport University of Sao Paulo Sao Paulo Brazil

**Keywords:** diabetes, resistance exercise, weight training, glucose, insulin resistance

## Abstract

**Background:**

Type 2 diabetes (T2D) is preceded by prediabetes, and these conditions place a great burden on patients and society. These conditions are significantly associated with poor glycemic control, which is improved by resistance exercise. It has been suggested that resistance exercise should be performed with a high degree of effort to improve glucose metabolism, but this is associated with negative psychological responses that might lead to lower long-term adherence.

**Objective:**

This study aimed to investigate the effect of the degree of effort during a resistance exercise session on glycemic control and psychological responses in individuals living with prediabetes or T2D.

**Methods:**

This study will be a crossover, 3-arm, randomized controlled trial. A total of 15 participants living with prediabetes or T2D will be thoroughly familiarized with 7 resistance exercises; afterward, they will perform 3 randomized experimental sessions, each lasting approximately 48 hours each, separated by at least 4 washout days. In 2 of these sessions, supervised resistance exercise will be performed, but the sessions will differ in the degree of effort in each set (high vs low) and will be equalized in terms of total weight lifted and session duration. For this, proximity to failure will be manipulated by changing the number of sets per exercise, the number of repetitions per set, and the resting interval between sets and exercises. Participants will also complete a sedentary (control) session, where they will not perform any exercise. In response to each session, psychological responses will be assessed (exertion, affect, enjoyment, self-efficacy, and discomfort). Glycemic control will be assessed by a continuous glucose monitoring device every 5 minutes, throughout the approximately 48 hours of each experimental session. Food and drink will be individually prescribed by a registered dietitian nutritionist and provided to participants, in order to control for the confounding effect of energy intake and diet composition. Physical activity levels will be assessed by accelerometry. Randomization will be done using the opaque, sequentially numbered envelopes technique. Participants and researchers will be blinded for continuous glucose monitoring and accelerometry data, and data will be analyzed by a blinded statistician.

**Results:**

This study has been funded, and data collection is expected to take place between June 2024 and May 2025. Final manuscript submission should happen by August 2025.

**Conclusions:**

The results of this project might encourage individuals living with prediabetes and T2D to engage in resistance exercise while better informing exercise specialists on how to best incorporate resistance exercise in their client’s or patient’s routine.

**Trial Registration:**

ClinicalTrials.gov NCT06208189; https://clinicaltrials.gov/study/NCT06208189

**International Registered Report Identifier (IRRID):**

PRR1-10.2196/63598

## Introduction

Type 2 diabetes (T2D) is a disease in which peripheral insulin resistance associated with pancreatic beta-cell dysfunction leads to chronically elevated blood glucose levels [1]. If left unchecked over long periods of time, high glucose levels lead to vascular complications such as increased risk of cardiovascular diseases, diabetic nephropathy, neuropathy, retinopathy, and lower limb amputation [2]. In 2021, a total of 0.7 million people died due to diabetes or its complications in the United States [2], placing diabetes-related deaths as the eighth leading cause of death. T2D is preceded by prediabetes, a condition where blood glucose levels are higher than normal, but not high enough to be considered T2D [3]. The risk of individuals living with prediabetics developing T2D at some point in their life is around 50% [4], and approximately 1 in 3 Americans aged 18 years or older are currently living with prediabetes. The number of people living with diabetes in the United States was 32.2 million in 2021, and it is expected to increase to 36.3 million in 2045 [2]. Diabetes represents a great economic burden to society, as diabetes-related costs in the United States in 2021 were US$ 379.5 billion [2]. Approximately 95% of all diabetes cases are T2D [5].

It is an indisputable fact that exercise is a cornerstone for the prevention and treatment of prediabetes and T2D [6,7]. Of note, even an acute bout of exercise has profound positive effects on glucose metabolism [8]. In fact, exercise training effects on improved insulin sensitivity may be lost in as little as 6 days after the last exercise session in individuals living with T2D [9,10], suggesting the positive effects of exercise on glycemic control can be largely attributed to the acute improvements observed in the hours or days after each exercise bout [11]. Thus, understanding the acute effects of exercise on glucose metabolism is important to optimize exercise prescription for the prevention and treatment of T2D. Although aerobic exercise is usually the default choice for T2D treatment and prevention [7], resistance exercise is also a viable option for physically inactive individuals living with prediabetes and T2D [12,13].

It has been repeatedly shown that an acute bout of resistance exercise improves insulin sensitivity and glycemic control during at least the following 24 hours in individuals living with prediabetes and T2D [11,14,15]. For example, van Dijk et al [11] assessed 24-hour glycemia with a continuous glucose monitoring (CGM) device after bouts of resistance or aerobic exercise in individuals living with prediabetes not on medication, in individuals living with T2D on oral hypoglycemic medications, and in individuals living with T2D on insulin. They reported that regardless of the condition, resistance and aerobic exercise equally reduced the prevalence of hyperglycemia and improved glycemic control. Thus, resistance exercise’s effects for preventing and treating prediabetes and T2D have been increasingly recognized, and prescription for muscle strengthening is highly recommended by international guidelines [7]. Despite this, while compliance with the current American College of Sports Medicine’s recommendations for muscle strengthening remains low in Americans, at ~27% [16,17], the proportion of individuals living with diabetes that meet strength training guidelines is even lower, at ~13% [18].

Resistance exercise prescription entails the manipulation of many variables. These include exercise type (single- or multijoint) and order, load, number of sets per exercise, repetitions per set, repetition duration (ie, tempo), and resting interval between sets [19-22]. There are also variables that are derived from the above, such as total volume-load (load × sets × repetitions), and session density (total volume-load / session duration). Appropriate manipulation of these variables is important for targeting specific adaptations [23]. However, the literature pertaining to the manipulation of these variables for the prevention and treatment of T2D is scarce. With this in mind, in a previous study [24-26], we assessed the effect of manipulating training volume by changing the number of sets in a session of resistance exercise in individuals living with obesity on insulin sensitivity. The data showed that a session composed of 21 sets improved insulin sensitivity, while a session composed of 7 sets did not (Silva et al, 2024, unpublished data).

More recently, attention has also been directed to the degree of effort during resistance exercise sets as a potentially important variable that can be manipulated to affect adaptative outcomes [27]. In fact, it has been suggested that a high degree of effort is important for acute improvements in glucose metabolism and insulin sensitivity in individuals living with T2D [28], although direct evidence of that is lacking. In our previous study [24-26], all sets were done with a high degree of effort (perceived exertion 9-10 out of 10 for all working sets). However, performing resistance exercise sets with a high degree of effort is associated with higher perceived exertion and discomfort [29], increased muscle soreness, negative psychological responses [30], and higher neuromuscular fatigue and muscle damage [31]. Taken together, these negative psychological and physiological responses to resistance exercise sets performed with a high degree of effort might negatively affect enjoyment and motivation during a resistance exercise session, which could ultimately reduce long-term adherence [32]. Individuals living with prediabetes and T2D might be more susceptible to these negative responses, as they are more physically inactive than the general population [33] and identify the lack of motivation and low self-efficacy (self-perception of the ability to accomplish something) as barriers to being more physically active [34].

Resistance exercise prescriptions should be tailored to meet not only the glycemic management needs of individuals living with prediabetes and T2D but also their motivation and self-perceived capacity to engage in exercise. Thus, the aim of this study is to assess the effects of the degree of effort during a resistance exercise session on glycemic control and psychological responses in individuals living with prediabetes and T2D. The hypothesis is that the degree of effort will not affect glycemic control, but that a high degree of effort will negatively affect psychological responses.

## Methods

### Description of Participants

Participants aged between 18 and 75 years will be recruited through posters and flyers at strategic points and by word of mouth in order to support diversity. More specifically, we will recruit from the University of New Mexico’s hospital, clinics, and facilities (both from the Main and North campuses), and from the Albuquerque area in New Mexico. After showing interest, potential participants will be thoroughly informed about the study’s risks and benefits, and they will be asked to answer a medical and physical activity history questionnaire in order to assess eligibility [35]. The inclusion criteria will be presence of prediabetes (fasting glycemia between 100 and 125 mg/dL or glycated hemoglobin [HbA_1c_] between 5.7% and 6.4%) or T2D (fasting glycemia 126 mg/dL or above or HbA_1c_ 6.5% or above), diagnosed by a medical doctor. Glycemic status will be confirmed by assessing a fasting blood sample. Exclusion criteria will be renal failure; liver disease; uncontrolled hypertension (>160 mmHg systolic and/or >100 mmHg diastolic); history of severe cardiovascular problems; inability to perform resistance exercise; being pregnant or trying to become pregnant during the course of the study; use of oral contraceptives; and in case of being treated with biguanides (metformin), being on them for less than 3 months. If eligible, before initiating participation, subjects will sign the informed consent form previously approved by the Institutional Review Board (IRB). Participants will be reimbursed for expenses incurred due to the research and resulting from it. Participation will be discontinued in case of withdrawal of consent or in case of injury or sickness (eg, skeletomuscular injury and having a cold). We will report reasons for withdrawal and discuss the reasons qualitatively. Adverse events related or nonrelated to the research protocol experienced by participants during the study will be carefully recorded and reported in the final manuscript.

### Study Type and Design

**Figure 1:**
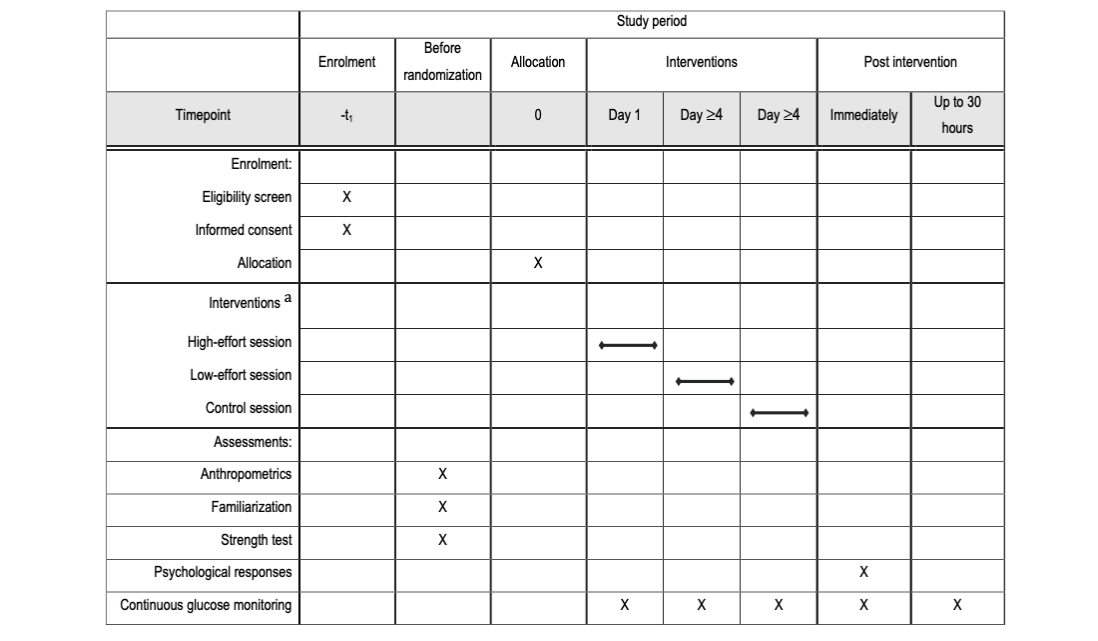
SPIRIT (Standard Protocol Items: Recommendations for Interventional Trial) schedule of assessments at different time points. a: Sessions order is randomized with at least a 4-day washout period.

This study will be a crossover, 3-arm, randomized controlled trial. The protocol description followed the SPIRIT (Standard Protocol Items: Recommendations for Interventional Trial) [36] and the TiDieR (Template for Intervention Description and Replication) [37] guidelines (Figure 1 and Multimedia Appendices 1-4). Upon meeting eligibility criteria and agreeing to participate, participants will undergo an anthropometrics assessment, familiarization sessions, and strength assessment before randomly carrying out 3 separate experimental sessions (2 exercise and 1 control), with each session taking place over ~48 hours, over 3 consecutive days. The order of the sessions will be randomized and they will be separated by a minimum of 4 washout days.

During experimental sessions, participants’ diets will be strictly controlled, and all food and drink will be provided to the participants. Glycemic control will be monitored using a CGM device throughout the experimental sessions. The exercise sessions will be matched for total volume load and session density, but proximity to concentric muscular failure (ie, incapacity to continue the set in the concentric phase of the movement) will be manipulated to vary the effort between the exercise sessions. The control session will follow identical procedures except that participants will remain sedentary for the same period of time they are to exercise in the exercise sessions.

Participants will be asked to refrain from moderate- to high-intensity physical exertion for 72 hours before the experimental sessions. The evening before the exercise or control sessions (day 1), at ~5 PM, participants will report to the laboratory to be instrumented with the CGM device and an accelerometer (to assess physical activity levels, refer to *Continuous Glucose Monitoring* subsection) and will receive dinner to be ingested at 6:30 PM and a snack to be ingested at 9:30 PM. The next morning (day 2), they will report fasted to the laboratory at 8:00 AM and ingest the provided breakfast at 8:30 AM. The exercise or control sessions will begin at 9:30 AM and will last ~50 minutes. They will perform 1 of 3 random sessions: a high-effort resistance exercise, a low-effort resistance exercise, or control session. After each session, psychological responses (perceived exertion, enjoyment, affect, discomfort, and self-efficacy) will be assessed.

After the session, participants will then be free to resume their daily activities after being given individualized meal bags and will be asked to consume lunch at 12:00 PM, a snack at 3:30 PM, dinner at 6:30 PM, and another snack at 9:30 PM, and in the following day (day 3), they will have breakfast at 8:30 AM, lunch at 12:00 PM, and a snack at 3:30 PM. After that, they will report to the laboratory between 5 and 6 PM to have the CGM device and accelerometer removed. Participants will be instructed to refrain from moderate- to high-intensity physical exertion until they are deinstrumented.

**Figure 2:**
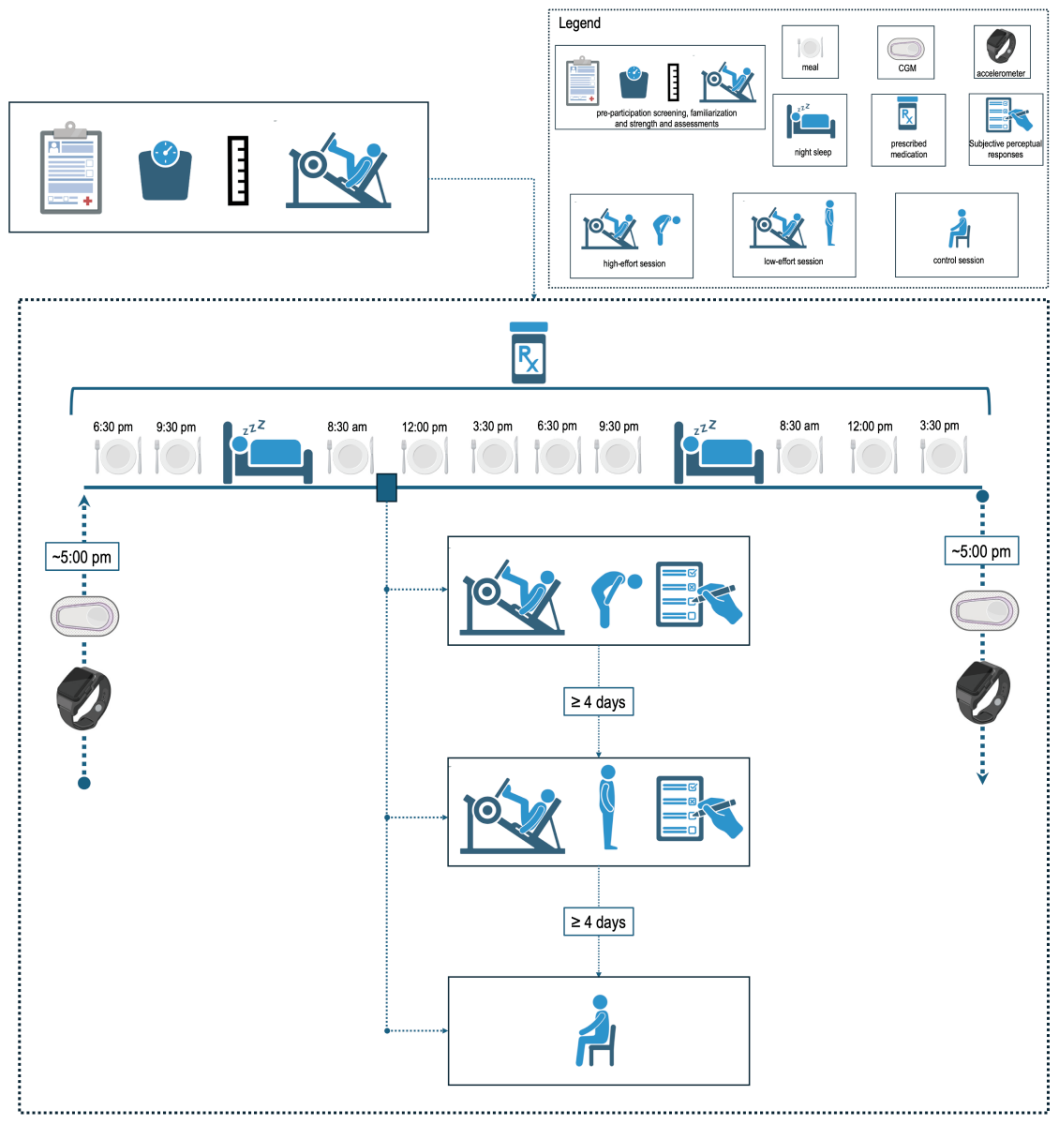
Study design. CGM: continuous glucose monitoring.

Throughout the entire study period, participants will be explicitly instructed not to alter their prescribed medication and to report any prescription changes made by their medical doctor during participation. Any adverse events whether related or nonrelated to the research protocol experienced by participants during the study (eg, skeletomuscular injury and having a cold) will be carefully recorded and reported in the final manuscript. Figure 2 depicts the study design.

### Anthropometrics

Body weight and height will be measured on an analog scale and a stadiometer, respectively, and BMI (kg/m^2^) will be calculated. Subsequently, the waist circumference will be measured with a measuring tape parallel to the ground over the umbilicus. Body composition will be evaluated using multifrequency bioelectrical impedance analysis (Bioelectrical impedance analysis, InBody 770) following manufacturer instructions.

### Familiarization and Strength Tests

**Figure 3:**
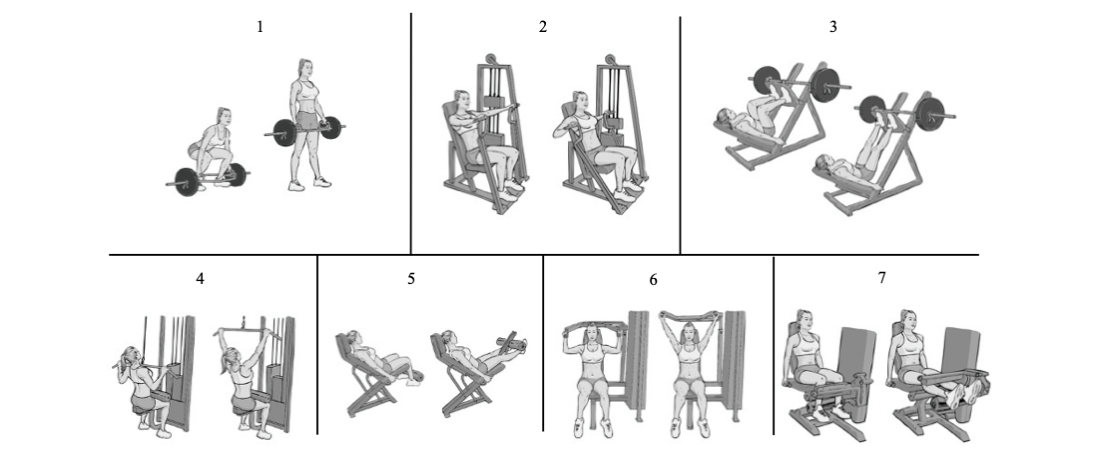
Exercises that will be prescribed for the strength exercise sessions: (1) hex bar squat, (2) seated chest press, (3) leg press, (4) lat pulldown, (5) leg extension, (6) shoulder press, and (7) leg curl.

Familiarization and strength tests will be performed as previously described [24-26]. Briefly, familiarization will consist of 5 sessions separated by at least 48 hours. In each session, participants will perform 3 sets of 8-10 repetitions of 7 exercises that target major muscle groups (hex bar squat, seated chest press, leg press, lat pulldown, leg extension, shoulder press, and leg curl; Figure 3), and load will be gradually increased throughout the familiarization sessions so that by the fifth session, participants will be able to perform the exercises with a high degree of effort (9-10 out of 10) based on the OMNI-Resistance Exercise scale [38]. Participants will also be familiarized with the psychological assessments during the familiarization period.

At least 72 hours after the fourth familiarization session, participants will perform the 10–repetition maximum (RM) tests [39,40] (the highest load one can lift for 10 complete repetitions with proper form) in each of the 7 exercises. After an initial 5-minute warm-up walking on a treadmill (~3-4 km/h, very light intensity) and 1 set with low load (12 repetitions, ~40%-50% 1RM, ~3-4 on the OMNI scale), the weight in each exercise will be adjusted to the weight used in the last familiarization session plus 5% to 10%, and participants will be instructed to attempt to perform 11 repetitions with good form (ie, with a full range of motion and tempo ~2:1 sec). If they perform the 11 repetitions with good form, the weight will be increased by 5% to 10%, and they will be given 180 seconds to recover and will make another attempt. Load will be increased until they can perform 10 repetitions or fewer with good form. If they perform fewer than 10 repetitions, the Brzycki equation [41,42] will be used to estimate participant’s 10-RM. All tests will be performed under the supervision of the same research team member and the load corresponding to the 10-RM in each of the exercises will be used in the exercise sessions (described in *Exercise and Control Sessions* subsection).

At least 72 hours after the strength assessments, participants will perform a fifth and final familiarization session. This familiarization session will be equal to the high-effort session (described below). Participants will perform the 7 exercises, 3 sets per exercise (total of 21 sets), aiming at 8 repetitions per set, using the load corresponding to their 10-RM. Participants will be asked to rate their exertion after each set using the OMNI scale, to ensure they are performing the sets with high effort (>8 on the OMNI scale). The resting period between exercises and sets will be 120 seconds. The main outcome from the fifth familiarization session is the number of repetitions the participant can perform in each set of the 7 exercises.

### Psychological Responses

After each set of each exercise, participants will be asked to rate their perceived exertion using the OMNI-Resistance Exercise scale from 0 to 10 [38]. During each session, participants will be asked to rank their feeling (before and immediately after) [43], enjoyment (immediately after) [44], self-efficacy (immediately after) [45], discomfort (5 min after the resistance exercise sessions) [46], and session rating of perceived exertion (5 min after the resistance exercise sessions) [29] using validated scales.

### Randomization and Blinding

After the fifth familiarization session, random allocation of the order of the experimental sessions’ participants will carry out will be performed as previously described [24-26]. The order in which the experimental sessions will be held will be randomized with the aid of the website. A collaborator not directly involved in any phase of the research (conceptualization, data collection and analysis, and reporting) will print the random orders generated by the program, insert them inside opaque, sequentially numbered envelopes, and seal them. After completing the fifth familiarization day, the envelope will be opened, and the sequence of experimental sessions will be revealed.

Due to characteristics inherent to this trial (resistance exercise), blinding of participants and researchers to exercise or control situations is not possible. However, participants and researchers will be blinded to CGM results (the blind model will be used). Furthermore, data and statistical analysis will be performed by a statistician blinded to groups and participants’ identities, as previously described [24-26].

### Exercise and Control Sessions

Participants will perform 2 exercise sessions and a control session, and the exercise sessions will be composed of 7 exercises (described above), both with the loads corresponding to 10-RM.

One of the exercise sessions will be a high-effort session (*high*), when participants will perform 3 sets per exercise (total of 21 sets) aiming at 8 repetitions per set. We anticipate participants will perform each repetition with a total duration of ~2.5 seconds. The resting period between exercises and sets will be 120 seconds. This will lead to a total session time of ~50 minutes.

The other session will be a low-effort one (*low*), when participants will perform 6 sets per exercise (total of 42 sets) but perform half of the number of repetitions of the *high* session (ie, 4 repetitions). A repetition duration of ~2.5 seconds and a resting period between exercises and sets of 60 seconds will also lead to a total session time of ~50 minutes.

Both the *high* and *low* sessions will have similar total volume-load and session density but differ in perceived effort after each set [47]. Pilot data from our laboratory in healthy untrained participants show that participants were able to complete both *high* and *low* sessions as prescribed, but average OMNI score after sets (8.5 vs 5.9), session rating of perceived exertion (7.5 vs 4), and perceived session discomfort (73 vs 40) were all higher in response to *high* sessions compared with *low* sessions.

For the control session, all procedures will be identical to the *high* session, with the exception of performing the resistance exercises. However, to simulate all other procedures participants will be positioned on the equipment for ~20 seconds for each of the 3 sets in each of the 7 exercises, but will not perform any repetitions, thus, performing sham sets.

### Heart Rate Assessment

To estimate cardiac work, heart rate will be assessed throughout the experimental sessions. Participants will be instrumented with a heart rate strap (Polar H10) and monitor (Polar V800), and their heart rate will be recorded continuously during the experimental situations. Data will be synced into Polar Flow and downloaded to a spreadsheet before being analyzed.

### CGM Assessment

Glycemic control will be assessed by a CGM device (Dexcom G6 Pro; Dexcom) every 5 minutes, over the ~48-hour experimental sessions. Reporting of variables will follow the most recent international guidelines consensus for clinical trials [48] and will include (1) time in range: 70-180 mg/dL; (2) time in tight range: 70-140 mg/dL; (3) time below range: <70 mg/dL; (4) time in level 1 hypoglycemia: 54-69 mg/dL; (5) time in level 2 hypoglycemia: <54 mg/dL; (6) time above range: >180 mg/dL; (7) time in level 1 hyperglycemia: 181-250 mg/dL; and (8) time in level 2 hyperglycemia: >250 mg/dL. Data will be reported as a percentage of time per day and as estimated hours and minutes per day. We will also report (9) average glucose concentration and (10) area under the curve, (11) glucose management indicator (used to predict long-term glucose exposure); (12) glycemia risk index (summary of the quality of glycemia), (13) glucose coefficient of variation; and (14) mean SD glucose (both are measures of dynamic glucose variability). Variables will be reported for the entire 48-hour collection period, for the first and second 24-hour periods, and stratified over 4 different periods: morning (6 AM to 12 PM), afternoon (12 PM to 6 PM), evening (6 PM to 12 AM), and nocturnal (12 AM to 6 AM) [11]. Individual data will be analyzed only if the percentage of data obtained is >70% of the anticipated amount of data collected during the collection period [48].

### Diet and Physical Activity Levels

During the ~48-hour experimental situations, diet prescription will follow American Diabetes Association recommendations [49,50] to ensure a personalized diet based on their estimated energy requirements. Meals and snacks will be provided in individualized, pre-weighted packages and will be composed of ~55% carbohydrate, ~15% protein, and ~30% fat. The timing of ingestion will be strictly controlled to ensure a standardized diet. Diet prescription will be done by a registered dietitian nutritionist. The diet will be designed to meet the energy requirements as calculated with the Cunningham equation [51] multiplied by a physical activity level of 1.2 [11].

Participants will be instructed to maintain their habitual physical activity levels, and to not perform moderate- to high-intensity physical activity 72 hours before and during the experimental sessions. Physical activity levels will be assessed using a commercially available accelerometer (GT3X, ActiGraph).

### Statistical Analysis

The sample size was calculated using data from relevant literature using similar individuals and glucose-assessing tools (CGM) [11,14,52,53]. Conservative parameters were inserted in the G*Power program (Heinrich-Heine-Universität Düsseldorf, version 3.1.9.6) for a repeated measure ANOVA within interaction: effect size of 0.2 (for 24-hour average glucose concentrations), probability of error type **α** of 0.05, power (probability of error type 1 β) of 0.95, correlation among repeated measurements of 0.7 [54], and nonsphericity correction of 0.5 [55,56]. With these parameters, 15 individuals are required (actual power of 0.96). Considering a typical dropout rate of 25% to 40%, we will initially recruit 25 participants.

A collaborator outside the research team will double-enter data coded for allocation into a computer in separate spreadsheets, allowing the researcher responsible for data analysis to assess the data without access to allocation information. Allocation will only be revealed after statistical analyses are run. In case of doubts about data entry (missing values or values outside the reference range), the researcher responsible for data analysis will contact the collaborator outside the research team responsible for double entry of the data to check accuracy. There will be no interim analysis.

To ensure confidentiality, all study-related information will be securely stored at the study site. All participant information will be kept in locked file cabinets in areas with access controlled by the principal investigator. All laboratory specimens, reports, data collection, process, and administrative forms will be identified by a coded ID number only to maintain participant confidentiality. Records containing names or other personal identifiers, such as locator forms and informed consent forms, will be stored separately from study records identified by code number. No personal information from participants will be released outside the study. Full access to the complete dataset will be restricted to members of the research team.

The statistical analysis will follow the principles of intention-to-treat analysis [57]. Thus, data from participants will be assessed as randomized, regardless of whether they received the randomized treatment, meaning that even if they fail to follow the physical activity and diet instructions, or fail to complete the sessions as required, their data will be included in the analyses. Furthermore, we will also analyze the data “per protocol” [58], meaning that participants who deviate from instructions will be excluded from the final analyses. Differences in results stemming from intention-to-treat and per-protocol analyses will be discussed accordingly. The available variables will also be compared between participants who withdraw from the study and those who complete the study.

The effect of resistance exercise on glycemic control will be calculated as the absolute and percentage change between control and resistance exercise situations. Data will be expressed as mean and SD, with a 95% CI. For the analysis of data normality, we will perform the Shapiro-Wilk test. For normally distributed data, ANOVA will be used with 1 source of variation (experimental condition). If a significant main effect is observed, the post hoc Tukey test will be conducted. For nonnormal data, the Kruskal-Wallis test or Friedman test will be used as appropriate. The effect size will be calculated and interpreted as follows: <0.2=no effect, 0.2-0.49=low effect, 0.5-0.79=medium effect, and >0.8=large effect [59,60]. The significance level is set at 5%. The Prism program (version 10.1.1, GraphPad Software) will be used to generate graphs and analyze results. This trial will be reported following the CONSORT (Consolidated Standards of Reporting Trials) guidelines [61-63].

### Data Monitoring Committee

A data and safety monitoring board will be composed of 2 medical doctors (a diabetes specialist clinician and a sports medicine specialist) and a biostatistician. The board will meet quarterly to address data and safety stemming from data collection. Specifically, they will analyze data related to adverse events related and unrelated to the study: musculoskeletal injuries (muscle strain, bruising, joint pain, back pain, etc), cardiovascular issues (increased blood pressure, arrhythmia, angina, myocardial infarction, etc), acute illnesses and symptoms (bronchitis, pneumonia, asthma attacks, flu, heart attacks, strep throat, respiratory infections sore throat, fever, cough, diarrhea, sneezing, headaches, etc), falls, syncope, aggravation of preexisting conditions (arthritis, tendinopathy, etc), hospitalizations, and death. The board will also analyze the dropout rate, as well as the reasons for dropping out. The board will make independent decisions and will have no conflict of interest regarding data collection, analysis, interpretation, and publication.

### Ethical Considerations

This study’s protocol and informed consent were submitted to and approved by the University of New Mexico IRB (protocol # 2310089095), and all procedures will follow the ethical standards of the IRB on human experimentation and the Helsinki Declaration of 1975, as revised in 2000. The study was prospectively registered in a public clinical trial registry [64]. Any modifications to the protocol will be submitted to the ethics committee, followed by an update of the trial registry. To promote participant retention, financial compensation (USD $200) for their time and effort will be provided.

For data sharing, all personal identifiers will be stripped from the data. Specifically, any direct identifiers (name, email addresses, phone, and cell phone numbers), date identifiers (birthday, date of disease diagnosis, and dates patients participated in data collection), location identifiers (personal or professional addresses and zip codes), links to external datasets identifiers (social media accounts) will be removed. Furthermore, participants will be represented by an ID string generated randomly, and their data will be inserted in the files in a random order, not associated with the order they were enrolled. Thus, data will be entirely deidentified before sharing, in order to protect sensitive personal information and privacy.

## Results

As of June 2024, we have performed pilot studies and recruited 2 potential participants. The anticipated data collection period will be from June 2024 to May 2025, and the final manuscript is expected to be submitted for publication by August 2025.

## Discussion

### Potential Impact and Significance of the Study

The results of this study will contribute to a better understanding of the effects of resistance exercise in individuals living with prediabetes and T2D and will be relevant to trainers and therapists who seek to incorporate resistance exercise in their client’s or patient’s routine. Specifically, understanding how short-term glycemic control is affected by altering resistance exercise prescription, in the case of this study, degree of effort, and assessing how this affects psychological responses bears clinical and practical relevance. We anticipate that glycemic control will be similarly improved independently of effort, but participants will experience more positive feelings after the low-effort session. As feelings experienced during a resistance exercise session may be predictive of long-term adherence, this would suggest that correct manipulation of the degree of effort during a resistance exercise session might confer better long-term outcomes for individuals living with prediabetes and T2D.

### Strengths and Weaknesses

This study will take advantage of the latest technology to track glycemic control using a CGM device and accelerometry to directly assess physical activity levels. These are much better approaches compared with other glycemic (handheld glucometers or the oral glucose tolerance test) and physical activity (self-reported questionnaires) assessments, which would increase the reliability of the data as well as its application to real-world scenarios. Also, strict dietary control will be used, and glucose-lowering medication will not be altered, in order to tease out the specific effects of resistance exercise on glycemic control. Finally, a blinded statistician not directly involved in data collection will analyze data, in order to minimize any bias and increase the significance of our findings.

In this study, we will try to anticipate participants’ performance in the high-effort trial by performing a fifth and final familiarization session. This is necessary in case the low-effort trial is randomized to happen before the high-effort one, as the number of repetitions during the low-effort session will be prescribed as half of the high-effort session. However, if the number of repetitions performed in the high-effort session is different from the fifth familiarization session, this could introduce error. Also, to increase the external validity and scope of our results, we will include both male and female participants within a wide age range, as there is evidence that sex and age affect glycemic control [65-74] and response to glucose-lowering drugs [75-79]. Finally, because it has been demonstrated that glycemic control is worsened during the luteal compared with the follicular phase in premenopausal women [80], menstrual cycle phase might be a confounding factor in the present protocol.

## Appendix

Multimedia Appendix 1

Multimedia Appendix 2

Multimedia Appendix 3 SPIRIT (Standard Protocol Items: Recommendations for Interventional Trial) 2013 checklist.

Multimedia Appendix 4 World Health Organization trial registration dataset.

Multimedia Appendix 5 Approved informed consent.

## Abbreviations

CGM: continuous glucose monitoring
CONSORT: Consolidated Standards of Reporting Trials
HbA1c: glycated hemoglobin
IRB: Institutional Review Board
RM: repetition maximum
SPIRIT: Standard Protocol Items: Recommendations for Interventional Trial
T2D: type 2 diabetes
TiDieR: Template for Intervention Description and Replication
