# Supplementary material for: The Effect of Effort During a Resistance Exercise Session on Glycemic Control in Individuals Living With Prediabetes or Type 2 Diabetes: Protocol for a Crossover Randomized Controlled Trial
Source: JMIR Res Protoc. 2024 Nov 5;13:e63598. doi: 10.2196/63598 (PMC11576611; doi:10.2196/63598)
Supplement: Multimedia Appendix 1 [file resprot_v13i1e63598_app1.pdf]

RESEARCH ALLOCATIONS COMMITTEE

Funding decision

Magalhaes: The effect of effort during a resistance exercise session on glycemic control: a randomized-controlled trial

Congratulations! This proposal was selected for a funding amount of **$10,000**

Notes from reviewers

Rating #1

Budget justification	2: Inadequate
Clarity of presentation	4: Very Good
Broader impacts	5: Exceptional
Methods, work plan, and potential for completion	4: Very Good
Significance and innovation	5: Exceptional

New Research / Creative Direction	3: Adequate
New projects that have been favorably peer-reviewed by an extramural funding agency but were not funded yet but have a strong probability for eventual funding	1: Poor
Fields with limited Extramural Funding	1: Poor

Comments

This project promises merit to the field and probably New Mexico, specifically. The significance and innovation is described quite well.

This does not appear to be significantly new research or field of research for this faculty member.

This is a large grant request. This sort of project would be eligible for many other funding sources, as indicated in the application. [size=3]The actual cost of the project (at this time) is $16,500; the remaining $6,500 will be request from COEHS. It's unclear whether the project assistant identified in the budget falls under a faculty or post-doc salary. Budget justification should be more specific.[/size]

The project has not yet received IRB approval. It does not appear that an IRB application has been filed yet.

Rating #2

Budget justification	4: Very Good
Clarity of presentation	4: Very Good
Broader impacts	5: Exceptional
Methods, work plan, and potential for completion	4: Very Good
Significance and innovation	5: Exceptional

New Research / Creative Direction	5: Exceptional
New projects that have been favorably peer-reviewed by an extramural funding agency but were not funded yet but have a strong probability for eventual funding	4: Very Good
Fields with limited Extramural Funding	4: Very Good

Comments

Applicant proposes to investigate the effect of intensity of effort on the efficacy of resistance exercise in treating prediabetic and type 2 diabetic patients. International guidelines at this point are ambiguous about the required level of effort.

Insufficient effort is likely ineffective, whereas peak effort may cause injury and discomfort and be more difficult for patients to adhere to. This study, then, proposes to establish the level of necessary effort to achieve desired results, while also hopefully being sustainable in the long term.

Study proposes to have participants recruited and anthropemetrically assessed and then to have them engage in three sessions: one high effort, one low effort, and one control (where they just sit on the machines but do not exercise). Participants will be eating a controlled diet during the 48 hour period associated with each test, and they'll be continuously monitored with a glucose monitor and accelerometer during those periods.

[size=3]Proposal is well-written and clear and appears to have high probability of success. [/size]
[size=3] [/size]

Rating #3

Budget justification	4: Very Good
Clarity of presentation	5: Exceptional
Broader impacts	5: Exceptional

Methods, work plan, and potential for completion	5: Exceptional
Significance and innovation	4: Very Good
New Research / Creative Direction	2: Inadequate
New projects that have been favorably peer-reviewed by an extramural funding agency but were not funded yet but have a strong probability for eventual funding	4: Very Good
Fields with limited Extramural Funding	3: Adequate

Comments

Nicely presented proposal that will test how exercise behavior can lead to reducing insulin values for pre-diabetic and diabetic individuals. The theory and methodology are well presented. Sample size of individuals is limited to 15 but since this is a preliminary study (for a future larger version), the small sample might be expected. Budget is appropriately designed to for a project of this sort. applicant has (or should have) additional funding from other sources (including start-up).This is not new research altogether and thus it is assessed less highly than others that do represent new research. It has a high likelihood of acquiring additional extramural funding in the future.
