# Supplementary material for: The Effect of Effort During a Resistance Exercise Session on Glycemic Control in Individuals Living With Prediabetes or Type 2 Diabetes: Protocol for a Crossover Randomized Controlled Trial
Source: JMIR Res Protoc. 2024 Nov 5;13:e63598. doi: 10.2196/63598 (PMC11576611; doi:10.2196/63598)
Supplement: Multimedia Appendix 2 [file resprot_v13i1e63598_app2.pdf]

COLLEGE OF EDUCATION
& HUMAN SCIENCES

Date: November 29, 2023
To: **Flavio de Castro Magalhaes**, **Assistant Professor, HESS**

From: Cari Hushman, Ph.D., Interim Associate Dean for Research and Distance Education CH

Re: COEHS Research Office Mini-Grants

Congratulations! On behalf of the COEHS Research Office, we are pleased to inform you that your individual research proposal, titled "*The effect of effort during a resistance exercise session on glycemic control: a randomized-controlled trial*", has been funded in the amount of $4,000.00.

Please work directly with Shared Fiscal Services in the processing of all funds. All budgeted items must be used specifically for the purposes stated in your individual research proposal and must be committed by November 30, 2024. Any deviation from this must be approved by the Associate Dean for Research and Distance Education. No timeline extensions will be allowed. Please note, where applicable, the disbursement of research funds is contingent upon IRB approval of your research proposal.

If you are presenting or publishing work supported through these funds, please use the following statement to acknowledge the funding source:

> "This project was supported by the College of Education & Human Sciences Research Office at the University of New Mexico. The views expressed are those of the author(s) and do not necessarily reflect the views of the funders."

Submit a brief final report at the end of your project via InfoReady using the following link: https://unm.infoready4.com/. Please make sure that you complete the final report within InfoReady under the Progress Reports tab by indicating how the award was used and any professional benefits that may be linked to the award (e.g., publication opportunities [including citations], presentations, invited research collaborations, etc.). Your eligibility to request future funds requires that you complete this final report. These results will be used to review future applications, and to report outcomes of the COEHS Research Office Mini-Grant Initiative.

The COEHS Research Office assumes your willingness to honor these requests by virtue of your application. If you have any questions, please contact Dr. Cari Hushman. Once again, congratulations and best wishes on your work.

cc: Kristopher Goodrich, Dean, College of Education & Human Sciences
Sarah Valles, Academic Operations Officer, COEHS
Dr. Glenn Hushman, Department Chair, HESS
Darlene Lucero, Financial Analyst, Shared Fiscal Services, Office of the Provost
Bob Robinson, Department Administrator, HESS

Here is feedback from the review committee regarding your proposal:

- Literature review and background/context of the study is clear, as are the objectives of the research project. Methods of data collection and analysis are articulated.
- This is a timely and relevant topic that deserves attention from researchers interested in supporting health
- The background seems solid. It isn't completely clear how the proposed study will relate to the goal of tailoring exercise interventions to individuals. I am also unclear how the two different experimental exercise sessions differ from each other, why the sedentary session is needed, and what comparisons will be made and what specific hypotheses will be tested. Power analyses are under specified. Overall, I think this has potential to inform a research program, but more detail should have been given on the study design and the specific hypotheses to be tested with less on the background.
- The area is clearly important. I'm a bit skeptical that the sample size will be adequate to make any inferences that could be useful for tailoring, which seems to be the ultimate goal. That said, its fairly ambitious given the dollar request.
