# Supplementary material for: The Effect of Effort During a Resistance Exercise Session on Glycemic Control in Individuals Living With Prediabetes or Type 2 Diabetes: Protocol for a Crossover Randomized Controlled Trial
Source: JMIR Res Protoc. 2024 Nov 5;13:e63598. doi: 10.2196/63598 (PMC11576611; doi:10.2196/63598)
Supplement: Multimedia Appendix 4 [file resprot_v13i1e63598_app4.docx]

**WHO Trial Registration Data Set**

| **Data category** | **Information** |
| --- | --- |
| **Primary Registry and Trial Identifying Number** | <https://clinicaltrials.gov/study/NCT06208189> |
| **Date of Registration in Primary Registry** | 2024-01-19 |
| **Secondary Identifying Numbers** | World Health Organization Universal Trial Number (UTN): U1111-1303-5124 |
| **Source(s) of Monetary or Material Support** | University of New Mexico |
| **Primary Sponsor** | University of New Mexico |
| **Secondary Sponsor(s)** | UNM Research Allocation Committee grant ((#CmdxfJ), the UNM COEHS Research Office Mini-Grants, the National Council for Scientific and Technological Development (CNPQ: Grant#407975/2018-7 and # 402091/2021-3) and by the Minas Gerais State Agency for Research and Development (FAPEMIG: Grant# APQ-00008-22). |
| **Contact for Public Queries** | Dr Magalhães: [fcm@unm.edu](mailto:fcm@unm.edu) |
| **Contact for Scientific Queries** | Dr Magalhães: [fcm@unm.edu](mailto:fcm@unm.edu) |
| **Public Title** | Is the Degree of Perceived Effort During Resistance Exercise Important for Improvements in Blood Glucose? |
| **Scientific Title** | The Effect of Effort During a Resistance Exercise Session on Glycemic Control in Individuals Living With Prediabetes or Type 2 Diabetes Mellitus: a Protocol for a Randomized-controlled, crossover, Clinical Trial |
| **Countries of Recruitment** | USA |
| **Health Condition(s) or Problem(s) Studied** | Prediabetes and type 2 diabetes mellitus |
| **Intervention(s)** | Active: High- and low-effort strength exercise session |
|  | Comparator: control day without exercise |
| **Key Inclusion and Exclusion Criteria** | Inclusion criteria: age between 18 and 65 years; presence of prediabetes (fasting glycemia between 100 and 125 mg/dL or glycated hemoglobin [A1c] between 5.7 and 6.4%) or T2D (fasting glycemia 126 mg/dL or above or A1c 6.5% or above).  Exclusion criteria: renal failure, liver disease, uncontrolled hypertension (>160 mmHg systolic and/or >100 mmHg diastolic), history of severe cardiovascular problems, in case of oral hypoglycemic drugs usage, being on them for less than 6 months, inability to perform resistance exercise, being pregnant or trying to become pregnant during the course of the study, use of oral contraceptives, prisoners, persons requiring a legally authorized representative, non-English speakers. |
| **Study Type** | Interventional |
|  | Allocation: randomized controlled, crossover, 3-arm, statistician-blind |
|  | Primary purpose: prevention and treatment |
| **Date of First Enrollment** | June 28th 2024 (actual) |
| **Sample Size** | 15 (fifteen) |
| **Recruitment Status** | Not recruiting |
| **Primary Outcome(s)** | Statistically significant difference among groups after treatments in the result of glycemic control assessed via a continuous glucose monitoring device |
| **Key Secondary Outcomes** | Psychological responses (perceived exertion, enjoyment, affect, discomfort, and self-efficacy) |
| **Ethics Review** | Approved (UNM Institutional Review Board - protocol # 2310089095). |
| **Completion date** | NA |
| **Summary Results** | NA |
| **IPD sharing statement** | All data will be available. For data sharing, all personal identifiers will be stripped from the data. |
