# Supplementary material for: The Effect of Effort During a Resistance Exercise Session on Glycemic Control in Individuals Living With Prediabetes or Type 2 Diabetes: Protocol for a Crossover Randomized Controlled Trial
Source: JMIR Res Protoc. 2024 Nov 5;13:e63598. doi: 10.2196/63598 (PMC11576611; doi:10.2196/63598)
Supplement: Multimedia Appendix 5 [file resprot_v13i1e63598_app5.pdf]

THE UNIVERSITY OF NEW MEXICO

The effect of effort during a resistance exercise session on glycemic control: a randomized-controlled trial
Consent to Participate in Research
January 2024

Purpose of the research: You are being asked to participate in a research project that is being done by Dr. Flavio de Castro Magalhaes, from the Department of Health, Exercise, and Sports Science at the University of New Mexico. The purpose of this research is to study the effects of resistance exercise with different degrees of effort on your glucose responses (what we call glycemic control) and psychological responses. You are being asked to join because you are either an individual with prediabetes or with type 2 diabetes mellitus, but none of the following applies to you: renal failure, liver disease, uncontrolled hypertension (>160 mmHg systolic and/or >100 mmHg diastolic), history of severe cardiovascular problems, in case you use oral hypoglycemic drugs, you are using them for less than 6 months, unable to perform resistance exercise, being pregnant or trying to become pregnant during the course of the study, use of oral contraceptives, prisoners, persons requiring a legally authorized representative, non-English speakers.

This consent form contains important information about this project and what to expect if you decide to participate. Please consider the information carefully. Feel free to ask questions before making your decision whether or not to participate. Your participation in this research is voluntary.

Key information for you to consider:

- This project aims to evaluate the effect of effort during a resistance exercise session on glycemic control and psychological responses.
- Participation in this project involves 16 visits: 1 for screening and eligibility; 5 for familiarization sessions, 1 for a strength testing session; and 9 for the experimental conditions.
- Participation in this project will take a total of 19-20 hours active time over a period of 5 weeks.
- The main risks of participation involve:
    - Blood draw: bleeding at the site, feeling of lightheadedness when the blood is drawn, and rarely, an infection.
    - Injury from performing resistance exercise.
    - Wearing the continuous glucose monitoring device.

What you will do in the project: All research will take place in the Exercise Physiology Laboratory located in Johnson Gymnasium, Room B143, at The University of New Mexico.

Visit 1 – (1) informed consent, (2) medical and exercise history questionnaires, (3) medical clearance, (4) blood draw, (5) pregnancy test (women of childbearing potential only), (6) anthropometric characterization (estimated time commitment ~90 min): In this first visit, you will come to the lab in a fasted state, and can choose to sign the informed consent form after being thoroughly explained about the procedures, risks and benefits. Then you will answer a medical and exercise history questionnaire in order to assess if you are eligible to participate in the study. After that, if deemed eligible, a medical doctor, a member of the research team, will evaluate you, including measuring your blood pressure, in order to make sure you are cleared to participate in the study. Then, 15 milliliters of blood (equal to 1 table spoon) will be drawn from a vein in your arm by a trained

research team member to confirm your clinical status as a person with diabetes or prediabetes. If you are a woman of childbearing potential, we will provide you a free pregnancy test (urine) and you will be asked to take it in a private bathroom. You will be asked to inform the result to the medical doctor in a private room, or if you cannot interpret the result yourself, you will be asked to show the result to the medical doctor that will explain the result to you. If you are pregnant by the urine pregnancy test, you will be suggested to schedule an appointment with your health care provider to confirm the pregnant status. In case you have high blood pressure that indicates that taking part on this study would place risk on your health, or if you are pregnant, you will be thanked and compensated ($10.00) for your time, but you will have to be excluded from the study and any data collected from data will be destroyed. After that, your body weight, height and waist circumference will be measured. Subsequently, your body composition will be evaluated with the equipment called Bod Pod. For that, you will need to wear a bathing suit and a swimming cap (provided to you). The Bod Pod resembles an egg-shaped chamber, and has a window for you to look out and communicate with the researcher, and air will move in the enclosed space where you sit. A button at your knee while you are inside of the Bod Pod will allow you to have the door of the Bod Pod open immediately, in case you feel any discomfort. The entire test takes 5 to 10 minutes. There are no physical risks associated with any of these measurements. **Time commitment during this visit is ~90 min.**

Visit 2 – Familiarization #1 (estimated time commitment ~60 min): In visit 2, you will start being familiarized with the research protocol, which includes the resistance exercises and psychological questionnaires. A research team member will show you how to perform the seven resistance exercises (hex bar squat, bench press, leg press, lat pulldown, leg extension, shoulder press, leg curl), and as you perform them constant feedback will be given in order to make sure you are performing them with the correct technique. In this visit, you will perform 3 sets of each exercise, 10 reps per set, and the time between sets and exercises will be 120 sec. You will rate your own effort using a 0-10 scale and your effort should be 3-4 out of 10 in this session, which is “somewhat easy”. After the exercises, you will answer how you felt about the session including enjoyment of the activity and how hard you felt you were working. You can skip any question that makes you uncomfortable at any time. **Time commitment during this visit is ~60 min.**

Visit 3 – Familiarization #2 (estimated time commitment ~60 min): In visit 3, you will perform the same procedures as the last visit, but the weight you will lift will be increased so they are considered by you as “somewhat hard” or between 5-7 out of 10. Number of sets, repetitions, rest time and psychological questionnaires will follow the description of the first familiarization session. **Time commitment during this visit is ~60 min.**

Visit 4 – Familiarization #3 (estimated time commitment ~60 min): In visit 4, you will perform the same procedures as the last visit, but the weight you will lift will be increased so it is considered by you as “hard” or between 7-9 out of 10. Number of sets, repetitions, rest time and psychological questionnaires will follow the description of the first familiarization session. **Time commitment during this visit is ~60 min.**

Visit 5 – Familiarization #4 (estimated time commitment ~60 min): In visit 5, you will perform the same procedures as the last visit, but the weight you will lift will be increased so it is considered by you as “extremally hard” or between 9-10 out of 10. Number of sets, repetitions, rest time and psychological questionnaires will follow the description of the first familiarization session. **Time commitment during this visit is ~60 min.**

Visit 6 – Strength assessments (estimated time commitment ~60 min): In visit 6, you will have your strength tested in all seven exercises. After a warm-up, the weight in each of the exercises will be

adjusted so you can perform up to 10 repetitions. Rest time between exercises will be 120 sec (2 minutes). Based on that, we can know what weight to use in the exercise sessions below. **Time commitment during this visit is ~60 min.**

Visit 7 – Familiarization #5 (estimated time commitment ~60 min): In visit 7, you will perform a final familiarization session. You will perform 3 sets in each of the seven exercises lifting the weight assessed in the previous session, for as many repetitions as you can. After each set, you will rate your own effort, and after the session, you will answer the psychological questionnaires, as you did in the other familiarization sessions. **Time commitment during this visit is ~60 min.**

Visit 8 – Instrumentation with the continuous glucose monitoring device and accelerometer, and meals (estimated time commitment ~30 min): In visit 8, you will come to the lab only to be instrumented with the glucose monitoring device and an accelerometer (which is a sensor that measures your movement). Instrumentation simply means equipping you with the devices you will need to wear during data collection. You will also receive the meals you are to ingest. **It is very important that you let the researchers know if you have any food allergies or sensitivities before this visit.** You will be asked to come to the lab around 5 pm and the continuous glucose monitoring (CGM) device will be attached to the back of your upper arm. This device measures the glucose right under your skin every 15 minutes for many days, so it is very insightful for assessing how your glucose behaves throughout the day. Inserting a CGM sensor isn't painful, but there is a small needle in the sensor and the feeling is described as a slight pinching sensation as the sensor slides under the skin, after which the sensor is comfortable and easy to wear. A small area (~6 cm^2, ~1 $in.^2$) of the back of your upper arm skin will be shaved, lightly sanded, washed with non-moisturizing, fragrance-free soap, cleaned with alcohol, allowed to fully dry, before applying the sensor. To guarantee stickiness, a hypoallergenic and latex-free over-bandage will be applied over the sensor. You should not remove this device without talking to Dr. de Castro or a research team member. It is important you wear it the whole time, even while you bathe or sleep. During the course of the study, we ask you to maintain your regular daily physical activity levels, but not to perform moderate-high intensity physical activity in the 48 h that precede and during the experimental conditions. To measure your physical activity levels, you will wear an accelerometer on your wrist. This tiny, waterproof unit weighs very little (11 grams, ½ ounce), and looks like a watch. There is no inconvenience in wearing it. You don't have to, but you can take the device off while you bathe, but not while you sleep. Because what and how much you ingest can interfere with your glucose results, we will provide a total of 10 meals for you to ingest during each experimental condition. These meals are to provide you with all nutrients you need and are based on the American Diabetes Association guidelines. In this visit, you will receive 2 meals, to be ingested at pre-determined times. It is very important you do not deviate from ingesting these meals at the pre-determined times, and do not eat or drink anything else. **Time commitment during this visit is ~30 min.**

Please, be advised that you will perform visits 9, 12 and 15 in a random order. This means that we cannot anticipate the order of the session, and this will be decided randomly (like by flipping a coin). After session 7 we will open an envelope that will contain the sequence of the sessions. So, even though this document describes the sessions in a particular order, you might end up performing the control session first (described in visit 15), followed by the high-effort session (described in visit 9), and finally perform the low-effort session (described in visit 12). What is important for you to know, is that performing the sessions in a random order does not change anything for you, but is important for the study protocol.

Visit 9 – High-effort exercise sessions, and meals (estimated time commitment ~150 min): In visit 9, you will arrive at the lab at 8 am fasted, and then will ingest the breakfast provided to you at 8:30 am.

Then, at 9:30 you will perform the same exercise session you did on visit 7. After each set, you will rate your own effort, and after the session, you will answer the psychological questionnaires. At ~10:30 am you will have finished the exercise session, and will be free to resume your daily activities. We will provide you with meals to be ingested at pre-determined times. It is very important you do not deviate from ingesting these meals at the pre-determined times, and do not eat or drink anything else. **Time commitment during this visit is ~150 min.**

Visit 10 – De-instrumentation of the continuous glucose monitoring device and accelerometer (estimated time commitment ~30 min): In the evening following visit 9, at ~5-6 pm, you will report to the lab to be de-instrumented, i.e., the CGM device and accelerometer will be removed. **Time commitment during this visit is ~15-30 min.**

Visit 11 – Instrumentation with the continuous glucose monitoring device and accelerometer, and meals (estimated time commitment ~30 min): In visit 11, all procedures described in visit 8 will be repeated. **Time commitment during this visit is ~30 min.**

Visit 12– Low-effort exercise sessions, and meals (estimated time commitment ~150 min): In visit 12, you will arrive at the lab at 8 am fasted, and then will ingest breakfast at 8:30 am. Then, at 9:30 you will perform a slightly different exercise session. You will perform the same 7 exercises, but there will be 6 sets per exercise (total of 42 sets), with ~half the repetitions in each set compared to the session in visit 9. All other procedures will be the same, though: you will rate your own effort, and after the session, you will answer the psychological questionnaires. At ~10:30 am you will have finished the exercise session, and will be free to resume your daily activities. We will provide you with meals to be ingested at pre-determined times. It is very important you do not deviate from ingesting these meals at the pre-determined times, and do not eat or drink anything else. **Time commitment during this visit is ~150 min.**

Visit 13 – De-instrumentation of the continuous glucose monitoring device and accelerometer (estimated time commitment ~30 min): In visit 13, all procedures described in visit 10 will be repeated. **Time commitment during this visit is ~15-30 min.**

Visit 14 – Instrumentation with the continuous glucose monitoring device and accelerometer, and meals (estimated time commitment ~30 min): In visit 14, all procedures described in visit 8 will be repeated. **Time commitment during this visit is ~30 min.**

Visit 15 – Control session, and meals (estimated time commitment ~150 min): In visit 15, you will arrive at the lab at 8 am fasted, and then will ingest breakfast at 8:30 am. Then, at 9:30 you will perform a "fake" exercise session. In this session, all procedures will be identical to session 9, with the exception of performing the resistance exercises. To simulate all other procedures, you will be positioned on the equipment, but will not perform any repetitions. At ~10:30 am you will have finished the control session, and will be free to resume your daily activities. We will provide you with meals to be ingested at pre-determined times. It is very important you do not deviate from ingesting these meals at the pre-determined times, and do not eat or drink anything else. **Time commitment during this visit is ~150 min.**

Visit 16 – De-instrumentation of the continuous glucose monitoring device and accelerometer (estimated time commitment ~30 min): In visit 16, all procedures described in visit 10 will be repeated. **Time commitment during this visit is ~15-30 min.**

It is very important that during the entire study period, you do not alter your medication routine prescribed by your medical doctor. Any change should only be done directly by your medical doctor that prescribed that medication and if they decide to change it, it is extremally important you report that to Dr. de Castro or a research team member as soon as the change is implemented.

Risks:
(1) Blood draw
There are risks involved in drawing blood from an arm vein which may include momentary discomfort at the site of the blood draw, possible bruising, redness, and swelling around the site, bleeding at the site, feeling of lightheadedness when the blood is drawn, and rarely, an infection at the site of the blood draw. Every attempt will be made to draw the blood sample while ensuring safety and comfort to you. To minimize risk, proper blood drawing techniques will be used, which will include a sterile environment, a trained technician wearing non-latex sterile protective gloves, and thorough cleaning of the site. After the blood draw, we will apply pressure to the punctured site to prevent bruising and the site will be covered to prevent infection. If you have had symptoms or fainting with blood draws in the past, we will ask you to lay down while we obtain a sample. A researcher will stay with you for at least 15 minutes to ensure you are symptom free.

(2) Pain/discomfort/soreness and limited range of motion from resistance exercise
The risk of experiencing pain/discomfort/soreness and limited range of motion from resistance exercise exists, especially for exercises you are not used to. These risks will be minimized by having you perform many familiarization sessions, during which the weight you lift will be carefully increased, and based on your perceived effort, and by allowing a few days between sessions to avoid any lingering effect from the preceding session.

(3) Injury performing resistance exercise
The risk of injury performing resistance exercise is very low, but to minimize this risk, you will be thoroughly instructed on lifting form, you will perform several familiarization sessions during which load will be progressively and carefully increased based on your own perception of effort, constant feedback on lifting technique will be provided, and all sessions will be supervised by a trained research member.

(4) Cardiovascular risk performing resistance exercise
Resistance exercise is safe for the cardiovascular system, and adverse cardiovascular events have not been documented. However, to minimize the risk of a cardiovascular event, you will be evaluated and cleared to participate by a medical doctor, and will be instructed not to hold your breath while lifting the weight and maintaining normal breathing as you perform the repetitions. In the highly unlikely case that a cardiovascular event does happen, the research members are certified on cardiorespiratory resuscitation and the Exercise Physiology Laboratory is equipped with emergency medical equipment and emergency procedures are in place.

(5) Risk wearing the Continuous Glucose Monitor (CGM) device
Wearing the CGM device is safe. The risk of infection will be minimized by thoroughly cleaning the application area before applying the device onto your skin. The manufacturer instructions indicate that the device should be removed before MRI, CT scan, X-ray, or diathermy treatment. In case of discomfort, bleeding, irritation, or any complaint related to the CGM device, please contact the PI or medical doctor in the research team immediately.

What will happen if I am injured or become sick because I took part in this study? If you are injured or become sick because of this study, any emergency treatment will be at your cost. The

University of New Mexico makes no commitment to provide free medical care or money for injuries to participants in this study. It is important for you to tell Dr. de Castro immediately if you have been injured or become sick because of taking part in this study. If you have any questions about these issues, or believe that you have been treated carelessly in the study, please contact the Office of the Institutional Review Board (IRB) at (505) 277-2644 for more information.

Benefits: There is no direct benefit to you to participate in this study. However, after the study ends, you will be given a copy of your body composition and glycemic results (both from your blood draw, and from the CGM) and their results will be explained to you. Indirectly, the results of this study will contribute to a better understanding of the effects of resistance exercise in individuals living with prediabetics and type 2 diabetes and will be relevant to trainers and therapists that seek to incorporate resistance exercise in their client's or patient's routine.

Confidentiality of your information: We will take measures to protect the security of all your personal information, but we cannot guarantee confidentiality of all research data. The University of New Mexico IRB that oversees human research may be permitted to access your records. Your name will not be used in any published reports about this project. You will be assigned a random number for the confidentiality of data. All data collected will use this number and not your name. All data will be stored on a password protected computer and only approved research team members and Dr. de Castro will be able to access said data. All hard copies of data will be secured under lock and key and only approved research team members and Dr. de Castro will be knowledgeable of your identity during interactions required for the study.

Use of your information for future research: All identifiable information (e.g., your name, email) will be removed from the information collected in this project. After we remove all identifiers, the information may be used for future research or shared with other researchers without your additional informed consent.

Payment: In return for your time and the inconvenience of participating in this project, you will be paid US$10.00 [gift card] for each visit, plus US$40.00 for completing the protocol. Compensation is considered taxable income. If you complete all visits, this will add up to $200.

Parking: If you drive to UNM to participate in this research, parking at the Cornell visitor parking right next to Johnson Center will be provided free of charge for you.

Right to withdraw from the research: Your participation in this research is completely voluntary. You have the right to choose not to participate or to withdraw your participation at any time. The researchers also reserve the right to terminate procedures at any time if the researcher becomes aware of participant discomfort, other physiological concerns during testing, failure to comply with protocols, or if the researcher deems it unsafe to continue for any other reason. If you withdraw your consent, and request that your data is not used, your request will be honored, and your data will be destroyed. Consent forms will be kept for a period of three years per IRB regulations.

Clinical Trials: A description of this clinical trial will be available on http://www.ClinicalTrials.gov, as required by U.S. Law. This Web site will not include information that can identify you. At most, the website will include a summary of the results. You can search this Web site at any time.

If you have any questions, concerns, or complaints about the research, please contact:

Prof. Flavio de Castro Magalhaes, Department of Health, Exercise, and Sports Sciences, Johnson Center, Room B143, University of New Mexico, Albuquerque, NM 87131. Phone: (505) 277-2664. Email: fcm@unm.edu

If you have questions regarding your rights as a research participant, or about what you should do in case of any research-related harm to you, or if you want to obtain information or offer input, please contact the IRB. The IRB is a group of people from UNM and the community who provide independent oversight of safety and ethical issues related to research involving people:

UNM Office of the IRB, (505) 277-2644, irbmaincampus@unm.edu. Website: http://irb.unm.edu/

CONSENT

You are making a decision whether to participate in this research. Your signature below indicates that you have read this form (or the form was read to you) and that all questions have been answered to your satisfaction. By signing this consent form, you are not waiving any of your legal rights as a research participant. A copy of this consent form will be provided to you.

I agree to participate in this research.

________________________________	________________________________	_______
Name of Adult Participant	Signature of Adult Participant	Date

Researcher Signature (to be completed at time of informed consent)

I have explained the research to the participant and answered all of their questions. I believe that they understand the information described in this consent form and freely consents to participate.

________________________________	________________________________	_______
Name of Research Team Member	Signature of Research Team Member	Date
